# Hermaphroditism

**Published:** 1894-07

**Authors:** 


HERMAPHRODITISM—Dr. G. Bergenzelli reports (*West Med. Rep.*) the case of a thirty-eight-year-old Italian in whom the hairs in the pubic region extend to the umbilicus, the whiskers are thick, the pelvis of male formation, and the external genitals exhibited a vulva with the labia majora interposed in the superior extremity by the base of a well formed penis six centim. long, increasing three centim. during erection. The glans is well formed, has a slit in its inferior part, ending in a cul-de-sac; the prepuce adheres to the nymphæ, a median raphe in the lower surface of the penis leads to the meatus and presents in its extremity Pozzi's frænum. The vagina is narrow, and digital examination reveals the presence of the uterine neck. Menstruation has been regular since its appearance at the age of eighteen years. This individual has had sexual relations both with male and female, has aborted twice, but has never impregnated any woman. The case is one of gynandrism with female predominance.—*Ex.*

